# Insights into the role of an Fe–N active site in the oxygen reduction reaction on carbon-supported supramolecular catalysts†

**DOI:** 10.1039/c9ra09301j

**Published:** 2020-02-28

**Authors:** Lin Gu, Yunyun Dong, Yan Zhang, Bo Wang, Qing Yuan, Hongmei Du, Jinsheng Zhao

**Affiliations:** Shandong Key Laboratory of Chemical Energy Storage and Novel Cell Technology, Liaocheng University Liaocheng 252059 P. R. China j.s.zhao@163.com; College of Chemistry and Chemical Engineering, Liaocheng University Liaocheng 252059 P. R. China dongyunyun@luc.edu.cn

## Abstract

In this study, a nitrogen-containing ligand supramolecule named PPYTZ was successfully synthesized using 2,6-pyridinedicarboxylic acid chloride and 3,5-diamino-1,2,4-triazole in order to carry out oxygen reduction reaction (ORR). Such a polymer provides abundant coordination sites for iron ions, and the PPYTZ–Fe/C composite catalyst was formed with the PPYTZ–Fe complex loading on the surface of Vulcan XC-72 carbon. The physical characteristics and ORR performance of the composite catalysts were characterized systematically *via* various relevant techniques, and their catalytic activity and reaction mechanism were evaluated and compared. The results showed that the catalytic activities and the reaction mechanism of ORR were highly dependent on the formation of an Fe–N unit. Accordingly, the PPYTZ–Fe/C catalyst containing Fe–N active sites exhibited high ORR catalytic activity (an onset potential of +0.86 V *vs.* RHE) in 0.1 M KOH. Such an Fe–N catalyst can accelerate the adsorption of O_2_ and increase the limiting current density (from 2.49 mA cm^−2^ to 4.98 mA cm^−2^), optimizing the ORR catalytic process from a two-electron process to a four-electron process (an *n* value of 3.8).

## Abstract 2

The PPYTZ–Fe/C catalyst containing Fe–N active sites exhibited high ORR catalytic activity and stability in alkaline media with a four-electron pathway progress.

## Graphical abstract

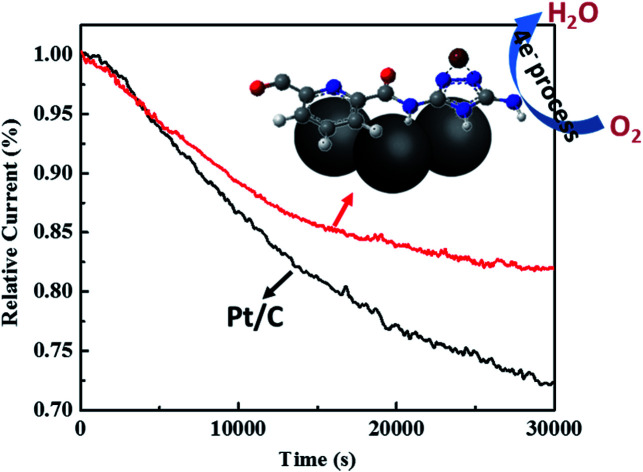

## Introduction

The massive consumption of fossil fuels has made environmental pollution and energy shortages a key issue. Therefore, the development of clean and efficient renewable energy technologies is an urgent task. In recent years, fuel cell technology has attracted considerable attention due to its high energy conversion efficiency and low pollution emission.^1–3^ However, the sluggish and complicated reaction process on the cathode of fuel cells leads to a larger overpotential, which might decrease the energy conversion efficiency of the cells.^4,5^ Platinum (Pt)-based catalysts and their corresponding cathode catalyst layers are considered to be a good option for the oxygen reduction reaction (ORR) catalyst, but its commercial applications are restricted owing to the high price and scarcity. Hence, reducing Pt loading and exploring the non-noble catalysts are two main approaches for reducing the catalyst cost.^6,7^

There are numerous reports about the cost reduction of Pt-free material catalysts, such as metal–organic frameworks (MOFs),^8,9^ inorganic nanoparticles,^10–12^ metal-free electrocatalysts,^13,14^ and non-precious metal electrocatalyst materials,^15–17^ in the last several decades. Among these, carbon materials have attracted increasing attention due to their high catalytic activity, low capital consumption, and outstanding durability.^18^ In particular, doping with nitrogen or, less commonly, other elements alter their (electronic) properties, making them suitable for applications as electrocatalysts for ORR. Moreover, the usual study suggests that the carbon-based materials containing N and metals, such as iron (Fe) and/or cobalt (Co),^19–21^ are alternative candidates as ORR electrocatalysts. Some nitrogen-rich pockets such as macrocyclic compounds, phenanthroline, pyridines, and N-containing heterocyclic polymers can coordinate with the metals easily. Jasinski *et al.* had found that cobalt phthalocyanine with a metal–N_4_ center can catalyze ORR in alkaline electrolytes.^22^ Yang *et al.* reported that the catalysts had closely comparable ORR activity, better durability, and methanol tolerance ability in comparison to Pt/C owing to the abundantly accessible Co–N_*x*_ active sites and fast charge transfer at the interfaces.^23^ Bao *et al.* showed a novel method for the development of highly active, durable oxygen electro-catalysts with Fe–N_*x*_, which is considered to be the most active species in ORR.^24^ Therefore, it can be inferred that the M–N–C (M = Fe, Ni, Co, *etc.*) type catalyst, which induces mass transfer and charge transfer behavior, could improve the dispersion of active species, conductivity, and O_2_ adsorption in the ORR process.

Nallathambi *et al.* found that the accessible active site density increased with increasing N/C ratio of the nitrogen precursor.^25^ To the best of our knowledge, the N/C ratio of 3,5-diamino-1,2,4-triazole (Hdatrz) was 2.5, which is larger than the most reported nitrogen precursors. Besides this, the molecular structures of the nitrogen precursor have a direct effect on the activity reaction mechanism of the ORR. In this work, we synthesized nitrogen-rich precursors with a plurality of pyridine rings and more than four chelating sites through polymerization between 2,6-pyridinedicarboxylic acid chloride and 3,5-diamino-1,2,4-triazole, and was named PPYTZ. The multiple chelating sites were beneficial to form an M–N_*x*_ active site, which should be responsible for the ORR activity of the resultant catalyst. We showed that the as-synthesized PPYTZ–Fe catalyst, prepared in this study, had good activity in the alkaline solution, and is a very good candidate as cathodic ORR catalysts for fuel cells.

## Experimental

### Materials

2,6-Pyridinedicarboxylic acid chloride and 3,5-diamino-1,2,4-triazole were obtained from Bailingwei Technology. Isopropanol (99.5%), *N*,*N*-dimethylformamide (DMF), KOH, K_3_[Fe(CN)_6_], and FeCl_2_ were purchased from Aladdin. Triethylamine, calcium chloride, and *N*-methylpyrrolidone (NMP) were purchased from Shanghai Kaiyin Chemical, Ltd. The carbon black (Vulcan XC-72) and Nafion (5 wt%) were obtained from Nanjing Hui Yu Energy Technology.

### Synthesis of PPYTZ, PPYTZ–Fe, PPYTZ/C and Fe–PPYTZ/C catalysts

**Scheme 1:**
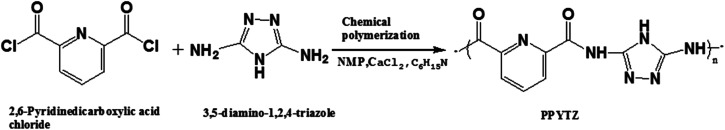
Synthetic route of PPYTZ.

**Fig. 1:**
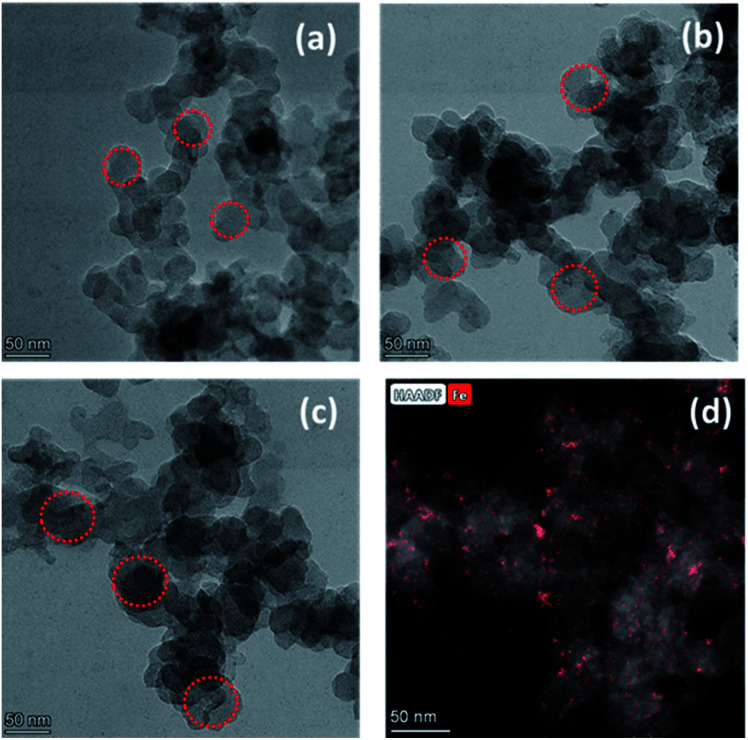
TEM images of (a) Vulcan XC-72, (b) PPYTZ/C, and (c) PPYTZ–Fe/C. (d) HAADF-STEM image and the Fe elemental mapping images of PPYTZ–Fe/C.

2,6-Pyridinedicarboxylic acid chloride (1.0 g, 4.9 mmol), 3,5-diamino-1,2,4-triazole (0.4857 g, 4.9 mmol), and 50 mL of *N*-methylpyrrolidone (NMP) were added to a 100 mL round bottom flask, and the mixture was stirred magnetically in an argon atmosphere at 95 °C. After 3 hours of the reaction, trimethylamine (15.0 mmol) and calcium chloride (9.0 mmol) was added to the system successively by means of a constant pressure dropping funnel. The above system was continuously stirred and refluxed for 48 hours. After the reaction, the synthesized material was washed with ethanol for three times. The resulting precursor was designated PPYTZ. Moreover, the structure of PPYTZ was characterized by the infrared-red spectrum and ^1^H NMR (Fig. 1S†). The synthetic route is shown in Scheme 1.

PPYTZ–Fe/C was synthesized through a simple refluxing and stirring process. Firstly, PPYTZ (100 mg) and FeCl_2_ (500 mg) were dispersed in 50 mL of DMF. The mixture was allowed to react under an argon atmosphere at 120 °C for 5 hours. Subsequently, it was cooled to room temperature, and 500 mg of Vulcan XC-72 carbon powder was added to the flask, followed by stirring for 24 hours. The activated carbon-supported catalyst was washed many times with water and dried. For comparison purposes, a metal-free catalyst named PPYTZ/C was obtained from mixing PPYTZ (100 mg) and Vulcan XC-72 (500 mg) in DMF (50 mL) for 24 hours.

### Characterizations

The surface topography characteristics were observed using field emission scanning electron microscopy (SEM) and transmission electron microscopy (TEM). The high-angle annular dark-field (HAADF) element mapping and energy-dispersive X-ray spectroscopy (EDS) were collected using a scanning tunneling electron microscopy (Tecnai G2 F20, FEI). Brunauer–Emmett–Teller (BET) measurement was conducted by the N_2_ adsorption/desorption analysis on an ASAP 2020 volumetric adsorption analyzer at 77 K. The X-ray diffraction (XRD) patterns were recorded on an XD-3 Purkinje diffractometer using Cu Kα radiation, and the scan rate was 5° min^−1^ over the range of 2*θ* = 10–60°. X-ray photoelectron spectroscopy (XPS) analyses were carried out on a Kratos Axis Ultra DLD electron spectrometer to analyze chemical elements and chemical configuration, and the binding energy was calibrated by means of the C 1s peak energy of 284.8 eV.

### Electrochemical characterization

The electrochemical measurements were carried out on an auto lab electrochemical workstation equipped with high-speed rotators from Pine Instruments (PINE Research Instrumentation) at room temperature. A typical three-electrode system was employed, including a catalyst-coated glassy carbon electrode (GCE) as the working electrode, a platinum foil as the counter electrode, and Ag/AgCl electrode in 3 M KCl solution as a reference electrode. The potential of the Ag/AgCl reference electrode was calibrated with respect to a reversible hydrogen electrode (RHE) by the formula of ERHE = EAg/AgCl + 0.197 V + 0.0591 pH in all the measurements.^26^ The oxygen reduction activities were evaluated using a rotating ring-disk electrode (RRDE) in a 0.1 M KOH as the test solution. The catalyst ink was prepared by dispersing 3.2 mg of the catalyst in 750 μL of the mixed solution containing water (570 μL), isopropanol (177 μL), and naphthol (3 μL). A homogeneous black suspension solution was obtained by ultrasonic treatment for 30 min. A calculated amount of the catalyst suspension was evenly deposited onto the clean GCE surface and dried at room temperature. All cyclic voltammograms (CV) were obtained at a scan rate of 100 mV s^−1^. The linear sweep voltammograms (LSV) were determined in an oxygen-saturated 0.1 M KOH solution with an electrode rotation rate ranging from 400 to 1600 rpm. The potential of the disk electrode was swept in the range from −1.00 V to +0.10 V, while the potential of the ring electrode was set at +0.500 V during the measurement.

## Results and discussion

### Structural characterization

**Fig. 2:**
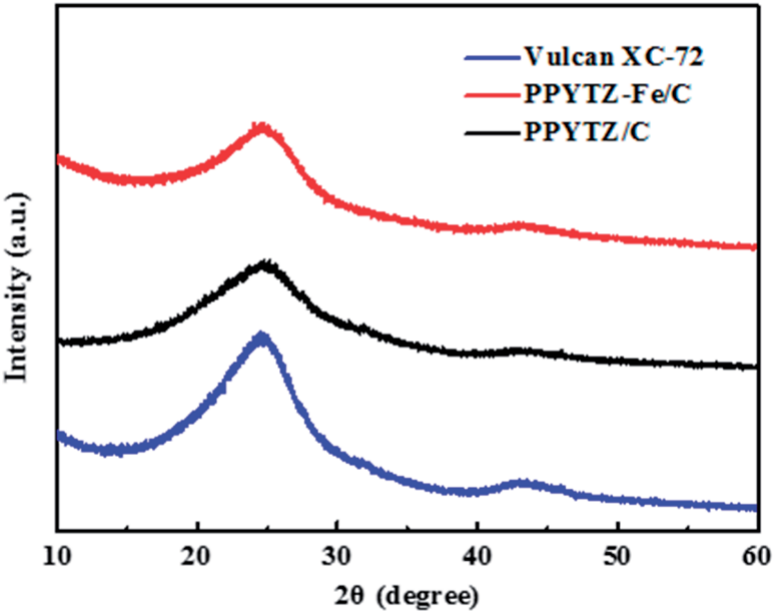
The powder X-ray diffraction (XRD) spectra of Vulcan XC-72, PPYTZ–Fe/C, and PPYTZ/C.

**Fig. 3:**
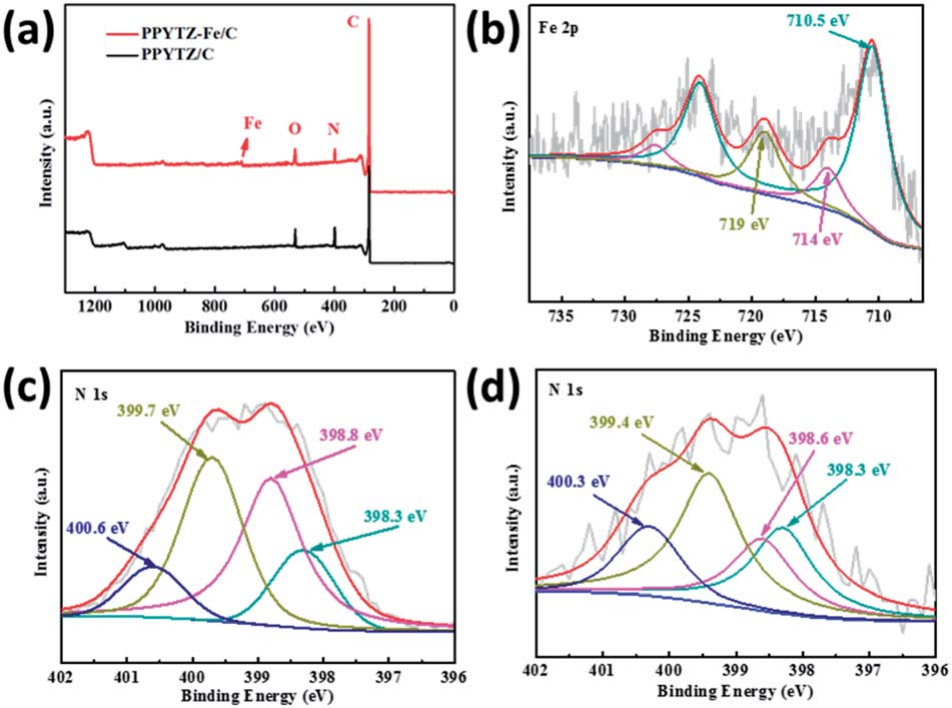
(a) X-ray photoelectron spectroscopy (XPS) of PPYTZ–Fe/C and PPYTZ/C, (b) Fe 2p peak of PPYTZ–Fe/C and N 1s peak of (c) PPYTZ/C and (d) PPYTZ–Fe/C.

The morphology of the resulting samples was observed by scanning electron microscopy measurements (SEM) (Fig. 2S†) and transmission electron microscopy (TEM). As shown in Fig. 1, the TEM observation reveals that all the catalysts show uniform sizes and better appearance for the microspheres. The TEM images of carbon black show a uniform particle size of 30–40 nm (Fig. 1a). For PPYTZ/C and PPYTZ–Fe/C catalysts, the catalysts were prepared by *in situ* polymerizations. It can be seen from the comparison between Fig. 1a–c that there were no obvious differences in the surface morphology between the catalysts, indicating that the polymer loading process does not have a large effect on the morphology of the catalyst. Intuitively, the particle size of PPYTZ/C and PPYTZ–Fe/C increases evidently than the untreated carbon powder, with the particle sizes being in the range of 50–60 nm and 60–70 nm for PPYTZ/C and PPYTZ–Fe/C, respectively. Such results indicate the formation of the composite catalysts. Thus, these results eventually will impart an important impact on the specific surface area and the porosity of the as-obtained catalysts. Moreover, the Fe content of the PPYTZ–Fe/C catalysts was performed by EDS, and the corresponding results are shown in Fig. 3S.† The results show that trace amounts of Fe signals (∼0.55 wt%) were detectable in EDS. In this case, the HAADF-STEM image and the Fe elemental mapping images of PPYTZ–Fe/C were provided (Fig. 1d), which showed that Fe really existed and was distributed uniformly in the samples.

**Fig. 4:**
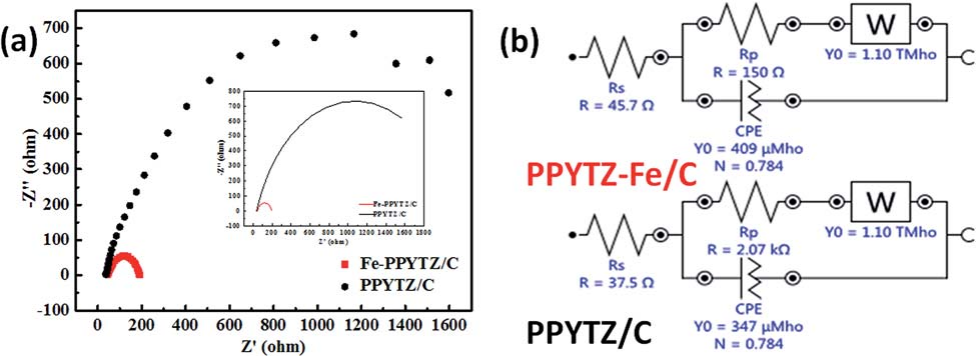
EIS (a) of PPYTZ/C and PPYTZ–Fe/C measured at a voltage of 0.85 V in a 0.1 M KOH solution at a frequency of 100 kHz to 0.1 Hz, (b) is an equivalent circuit diagram of EIS.

To further understand the porosity of these catalysts, their N_2_ adsorption/desorption isotherms and the pore size distributions were obtained. As shown in Fig. 4S,† all the cases exhibit typical type III adsorption isotherms, which suggest the presence of porous carbon-based adsorption materials. As compared in Fig. 4S,† the samples prepared with polymerization reactions demonstrated increasing areas of hysteresis. Moreover, it is obvious that the mesopore ratio of PPYTZ/C and PPYTZ–Fe/C was higher than that of the carbon precursors. The detailed specific surface area and the pore volume for each sample are given in Table 1. The pore volume values of PPYTZ/C and PPYTZ–Fe/C were found to be 0.24 cm^−3^ g^−1^ and 0.28 m^−3^ g^−1^, respectively, with the values exhibiting a larger pore size and pore volume due to the formation of some larger particles by the metal ion-introducing complex.

**Table 1:** Texture parameters of the alumina as determined by N_2_ adsorption–desorption isotherms

Samples	*S*_BET_ a (m^2^ g^−1^)	*V*_p_ b (cm^3^ g^−1^)	*d* c (nm)
Vulcan XC-72	189	0.56	1.9
PPYTZ/C	82	0.24	10.1
PPYTZ–Fe/C	91	0.28	12.9

a Specific surface area.

b Pore volume.

c Integrable pore diameter.

In order to determine the formation of the PPYTZ/C and PPYTZ–Fe/C catalysts, the material was subjected to X-ray diffraction (XRD) to obtain a diffraction pattern. Fig. 2 shows the XRD diagram of Vulcan XC-72, PPYTZ/C, and PPYTZ–Fe/C, respectively. For Vulcan XC-72, XRD reveals two broad peaks at 24.7° and 43.4°,^19,27^ which correspond to the diffraction peaks of graphite. For the PPYTZ/C and PPYTZ–Fe/C catalysts, the above two peak positions were almost the same, and no other detectable peaks that were different from the carbon powder in the XRD pattern of PPYTZ–Fe/C were observed, thereby indicating that iron ions(ii) were incorporated into the carbon framework, which suggested the intact amorphous nature of the carbon powder after being coated with the PPYTZ–Fe complex.

### Surface chemical state analysis

The high-resolution XPS measurements for PPYTZ–Fe/C and PPYTZ/C were performed to examine the surface chemical state. Compared to the PPYTZ/C catalyst, a distinct iron peak can be observed in PPYTZ–Fe/C (Fig. 3a). The spectrum clearly confirms that iron ion(ii) was successfully introduced into the PPYTZ polymer to form the PPYTZ–Fe/C catalyst. Furthermore, the chemical states of Fe 2p in the PPYTZ–Fe/C catalyst were investigated, as shown in Fig. 3b. After fitting analysis, the XPS spectra of Fe 2p can be deconvoluted into two peaks. The peaks at 710.5 and 714.0 eV can be assigned to the binding energies of the 2p_3/2_ orbitals of Fe^2+^ and Fe^3+^ species, respectively. Specifically, the peak at higher binding energies (BE) *i.e.*, 714.0 eV, can be assigned to the oxidation of Fe^2+^ ion. The peak at 710.5 eV suggests the presence of the Fe^2+^ ion coordinated with the ligand and the formation of the chemical bonding between the Fe and N moieties in the doped material.^28,29^ Meng *et al.* suggested that the metal ion center could promote the adsorption behavior of the intermediates, thereby maximizing the ORR activity.^18^ Furthermore, the nitrogen element also plays an important role in the ORR process of the catalyst. As shown in Fig. 3c, for the N 1s XPS spectrum of PPYTZ/C, it can be deconvoluted into four peaks: pyridine N at 398.5 eV, imine (–N=) at 398.8 eV, pyrrole-like N at 399.7, N atoms bonded to the triazole ring at 400.6 eV.^30,31^ The existence of N atoms in the triazole ring pyridinic N is reported to be highly related to the activity of the catalysts towards oxygen reduction. In contrast, for PPYTZ–Fe/C catalyst after introducing iron, the peaks corresponding to the N atoms in the triazole ring and amine N bonded to the triazole ring were observed at lower binding energy of 398.6 eV, 399.4 eV, and 400.3 eV, respectively, which may be the result of Fe–N active sites as demonstrated in the former literature.^19^

### Electrochemical impedance spectroscopy of the catalysts

The electrochemical impedance spectroscopy (EIS) was used to study the conductivity of the composite materials with the aid of various theoretical models.^32,33^ As shown in Fig. 4, the Nyquist plots of two catalysts and the corresponding circuit diagram was fitted and presented. The diameter of the semicircles, which represents the charge transfer resistance (*R*_p_) of the catalysts, was 2.07 kΩ for PPYTZ/C and 150 Ω for PPYTZ–Fe/C. It is obvious that the former was much higher than that of the latter one, indicating that PPYTZ–Fe/C exhibits better electron conductivity and lower charge transfer resistance during the electrochemical reaction after the introduction of the metal ions. Combing with the above BET analysis, PPYTZ–Fe/C had a higher specific surface area, higher pore volume, and higher conductivity than that of PPYTZ/C, which is expected to improve the catalytic performance of ORR.

### Performance of ORR

**Fig. 5:**
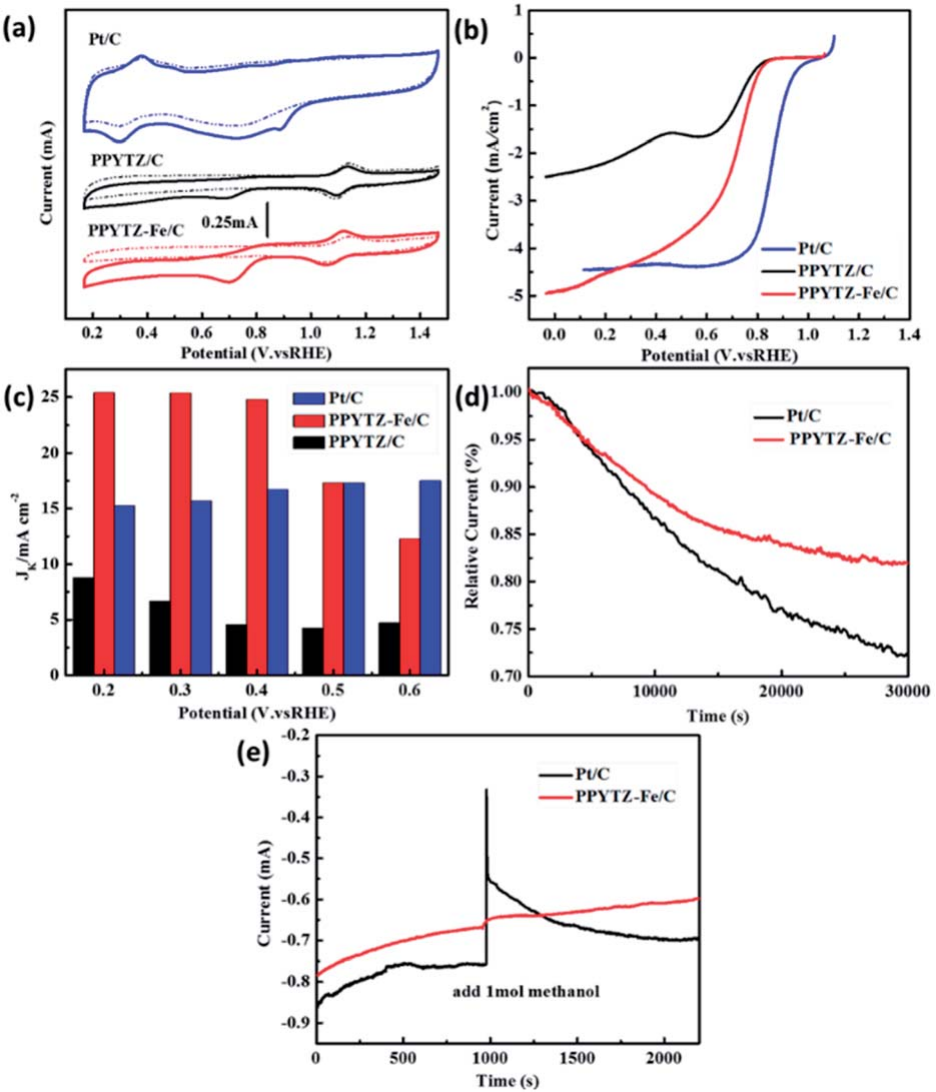
(a) CV curves of PPYTZ/C, PPYTZ–Fe/C, and Pt/C, (b) RDE voltammograms at 1600 rpm in O_2_-saturated 0.1 M KOH solution, (c) the kinetic limiting current density (*J*_k_) calculated at different potentials for PPYTZ/C, PPYTZ–Fe/C, and Pt/C, (d) long-term stability test of PPYTZ–Fe/C and Pt/C in 0.1 M KOH solution, (e) the methanol tolerance of PPYTZ–Fe/C and Pt/C in 0.1 M KOH solution.

The ORR performance of PPYTZ/C and PPYTZ–Fe/C samples was evaluated by cyclic voltammetry (CV) measurements in O_2_/N_2_-saturated 0.1 M KOH solution with a commercial Pt/C of 20 wt% Pt as a reference catalyst. As shown in Fig. 5a, in comparison with the electrochemical response in the N_2_-saturated solution, an apparent larger cathodic peak at around 0.70 V *versus* RHE was observed for the catalysts in the O_2_-saturated KOH solution for the PPYTZ/C and PPYTZ–Fe/C catalysts, which is inferior to Pt/C (0.89 V). The above phenomenon indicates that the presence of ORR processes in the catalyst electrode modification layers. Moreover, the peak current of Fe–PPYTZ/C increases sharply in an oxygen atmosphere to 0.45 mA compared with 0.23 mA in PPYTZ/C. The addition of iron significantly increases the peak current, which is an ideal property for high-performance ORR catalysts.

The activities were further evaluated on a rotating disk electrode (RDE) at a rotation of speeds of 1600 rpm with a scan rate of 10 mV s^−1^. As shown in Fig. 5b, the initial ORR potential (*E*_onset_) for both PPYTZ/C and PPYTZ–Fe/C was 0.87 V *vs.* RHE, which is very close to Pt/C (0.99 V *vs.* RHE). Notably, after the introduction of Fe ions, the limiting current density of the Fe–PPYTZ/C catalyst was 4.98 mA cm^−2^, which was twice than that of PPYTZ/C (2.49 mA cm^−2^), indicating that the Fe–N active site increases the limiting current density of the catalyst.^34,35^ In addition, the kinetic limiting current density (*J*_k_) was obtained through the intercept of linearly fitted Koutecky–Levich (K–L) plots at a potential range from 0.2 to 0.6 V *vs.* RHE (Fig. 5S†). The *J*_k_ was achieved to 25.2 mA cm^−2^ at 0.2 V *vs.* RHE for PPYTZ–Fe/C, which was much higher than that of PPYTZ/C (7.6 mA cm^−2^) and Pt/C catalyst (15.1 mA cm^−2^). It illustrates that introducing Fe–N active sites accelerates the ORR kinetics.

**Fig. 6:**
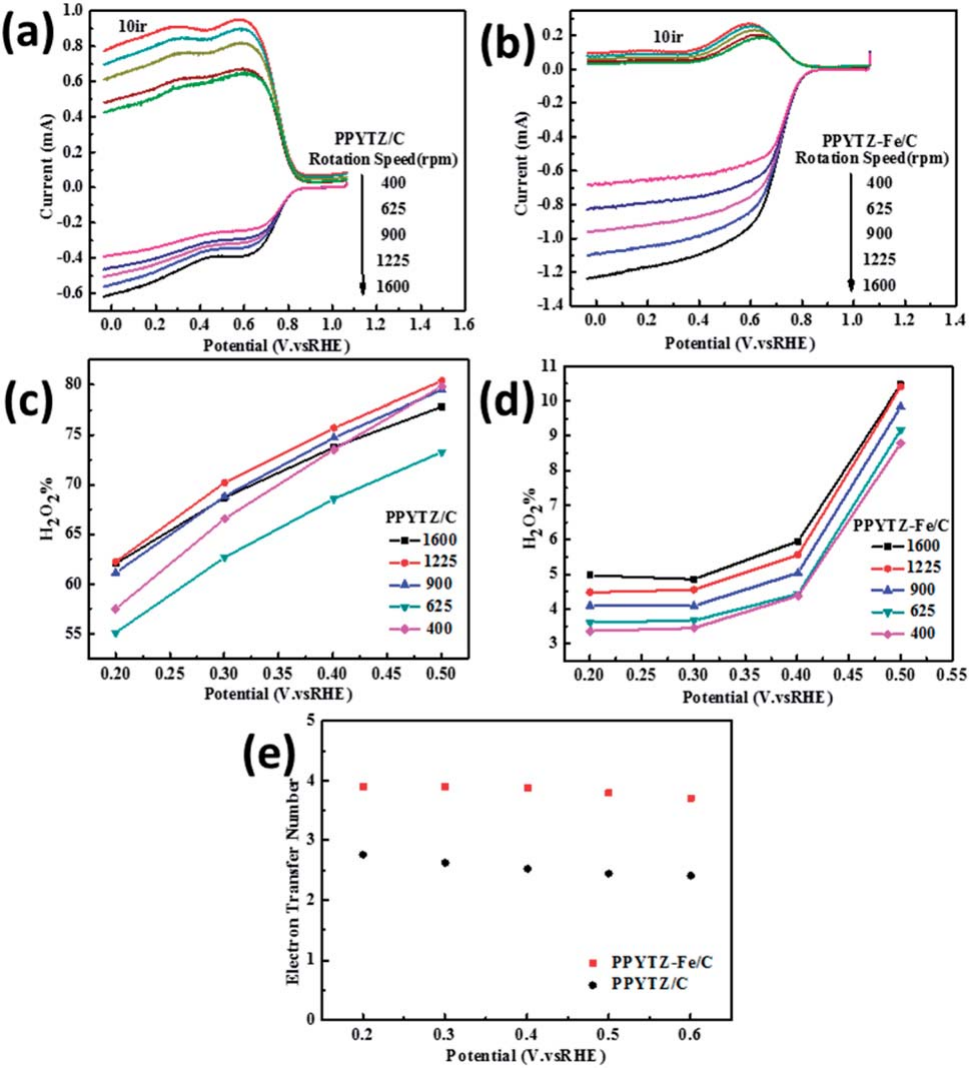
RRDE voltammograms for (a) PPYTZ/C and (b) PPYTZ–Fe/C. H_2_O_2_ yield of (c) PPYTZ/C and (d) PPYTZ–Fe/C at different speeds. (e) Electron Transfer Number (ETN) of PPYTZ/C and PPYTZ–Fe/C at different potentials.

The stability of the catalysts is also a significant parameter for evaluating the electrochemical performance of the catalyst. The stability of PPYTZ–Fe/C and PPYTZ/C for ORR was examined by continuous cycling with 5000 cyclic voltammetry cycles (Fig. 6S†). The results show that the voltammetric behavior of the PPYTZ–Fe/C catalyst differs smaller than that in PPYTZ/C. PPYTZ–Fe/C can retain stable catalytic activity after 5000 cycles, with the peak current reducing from 0.45 mA to 0.39 mA (13.3% loss). For the PPYTZ/C catalyst, the peak current was reduced from 0.23 mA to 0.16 mA, with the retention of only 86.96% of its original peak current (loss of 69.6%). The chronoamperometric testing was carried out to confirm the durability of the nitrogen-doped carbon capsules and commercial Pt/C in 0.1 M KOH. The results shown in Fig. 5d reveals that the PPYTZ–Fe/C sample retains *ca.* 80.0% of the initial current after 30 000 s, whereas Pt/C retains only 73.0% of the initial current. Moreover, the tolerance to methanol crossover was tested with the same measurement except for the addition of 1.0 M methanol at 1000 s. As can be seen in Fig. 5e, there is no noticeable change in the current density of PPYTZ–Fe/C catalyst, while a strong current response occurs at Pt/C catalyst, indicating that PPYTZ–Fe/C exhibits extremely good ability to resist the crossover effect. All these results demonstrate that the ORR activity of PPYTZ/C was greatly improved through the introduction of Fe species.

### Mechanism of ORR

RRDE test was used to further confirm the number of electrons transferred by the oxygen reduction reaction and calculate the corresponding H_2_O_2_ yield during the oxygen reduction reaction from eqn (1) and (2). The test was carried out in an O_2_-saturated 0.1 M KOH solution with a sweep rate of 10 mV s^−1^, a rotational speed of 400 rpm to 1600 rpm, a ring voltage fixed at 0.5 V (*vs.* Ag/AgCl).^36^ The reactants were first oxidized on the surface of the disc, and the oxidation product was rapidly transferred to the ring by the rotation of the electrode. The amount of reduction of H_2_O_2_ on the ring disk is represented by the ring current. For calculating the ring current, the equation used is as follows:1
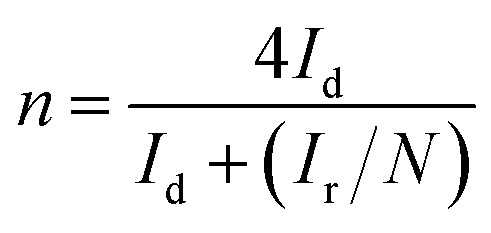
2
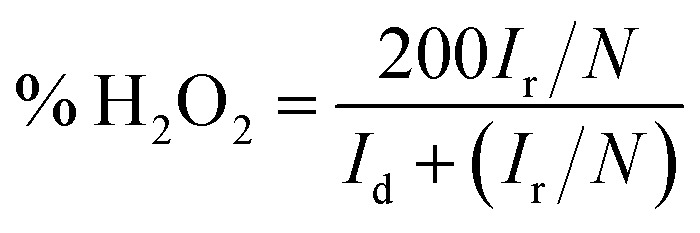
where *I*_r_ is the ring current (mA cm^−2^), *I*_d_ is the disk current (mA cm^−2^), and *N* is the collection efficiency (*N* is a constant, *N* = 0.37).^37^

The curve in Fig. 6 contains the upper and lower parts: the upper part of the figure shows currents of the ring electrode, while the lower part of the figure shows the disk current. The ring current value increases with the increase of the rotation speed, which means that the amount of H_2_O_2_ was detected to increase. Moreover, the ring current of PPYTZ/C (Fig. 6a) was significantly higher than the ring current of PPYTZ–Fe/C (Fig. 6b), which is much similar to RDE, indicating that the limiting current density (1.2 mA) of Fe–PPYTZ/C was about twice than that of PPYTZ/C (0.62 mA). Moreover, the initial potential of PPYTZ/C and Fe–PPYTZ/C was 0.87 V *vs.* RHE.

**Fig. 7:**
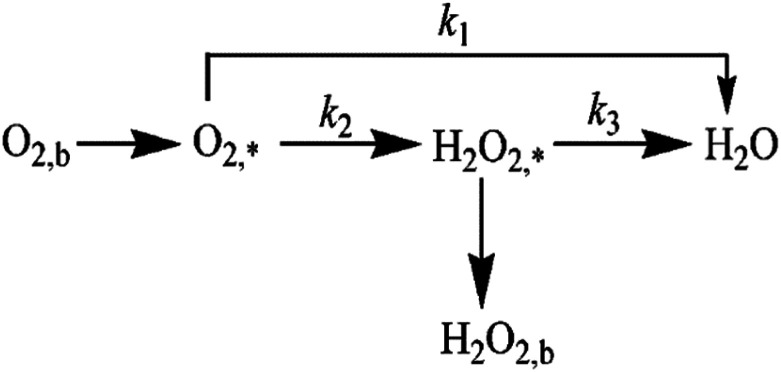
Graphical representation of direct 4e and indirect 2e + 2e pathways involving ORR on catalysts.

Subsequently, the production of hydrogen peroxide and the number of electron transfer was calculated in the voltage range of 55.1–79.4% for PPYTZ/C at the corresponding voltage (Fig. 6c). The electron transfer number was calculated from 0.2 V to 0.5 V *vs.* RHE. As shown in Fig. 6d, the hydrogen peroxide yield of PPYTZ–Fe/C catalyst was found to be 3.3–10.4%, which was lower than that in PPYTZ/C (55.1–79.4%) at the corresponding voltage (Fig. 6c). The electron transfer number calculated from the RRDE data at 1600 rpm (Fig. 6e) showed that the average electron transfer number of PPYTZ–Fe/C was 3.8, while the electron transfer number of PPYTZ/C was 2.7. Such results are consistent with the RDE data. The Fe–N sites can efficiently decrease the formation of the hydrogen peroxide sharply, and the ORR reaction was closer to the four-electron process, which indicates that Fe–N can accelerate the adsorption of oxygen. Table 2 also lists the ORR properties of other M–N catalysts. It was found that PPYTZ–Fe/C is an excellent oxygen reduction catalyst and is highly competitive on commercial roads (Fig. 7).

**Table 2:** Electrocatalytic performance of ORR for platinum-free catalysts reported in recent years

Catalyst	*E*_onset_ (V)	ETN	H_2_O_2_ (%)	Limit current (mA cm^−2^)	Electrolyte solution	Reference electrode	Loading (mg cm^−2^)	Ref.
PPYTZ/C	0.86	2.7	<79.4	2.49	0.1 M KOH	RHE	0.185	This work
PPYTZ–Fe/C	0.86	3.8	<10.4	4.98	0.1 M KOH	RHE	0.185	This work
Fe–NMCSs	1.027	3.94–3.99	<3.5	5.00	0.1 M KOH	RHE	0.255	18
NCNP-3	−0.143	2.96–3.12	<50	3.39	0.1 M KOH	Ag/AgCl	0.12	38
FeNC-900	−0.025	≈4	<10	1.50	0.1 M KOH	Ag/AgCl	0.125	19
Fe–P-900	0.95	3.62	1.3	5.01	0.1 M HClO_4_	RHE	0.53	39

The specific insights into more detailed oxygen reduction route data can also be acquired *via* RRDE measurements. Then, the ORR mechanism of the PPYTZ–Fe/C catalyst can also be understood semi-quantitatively. Damjanovic *et al.* proposed mechanisms for the four-electron transfer process.^40^ There are two possible transfer pathways: ① the direct four-electron pathway expressed as eqn (4) (*k*_1_ ≫ *k*_2_, *k*_3_ = 0); ② the “step-by-step” four-electron pathway expressed as eqn (5) and (6) (*k*_2_ = *k*_3_, *k*_1_ = 0). It should be highlighted that the rate reaction constants *k*_1_, *k*_2_, and *k*_3_ are the rate constants for each step, and calculated according to the eqn (8)–(10) to identify the exact mechanism of ORR of the PPYTZ–Fe/C catalyst.3
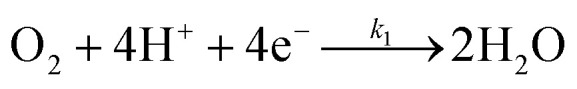
4
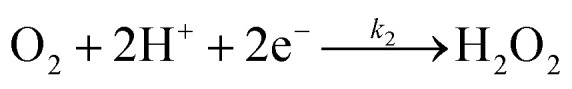
5
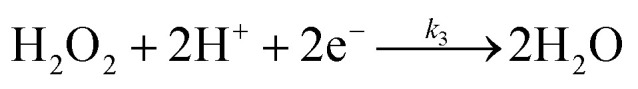
6
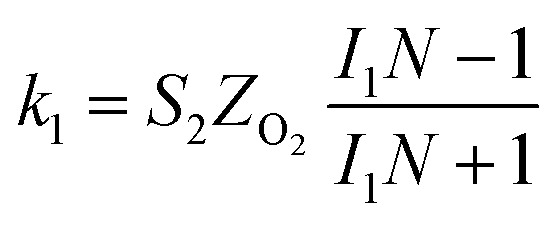
7
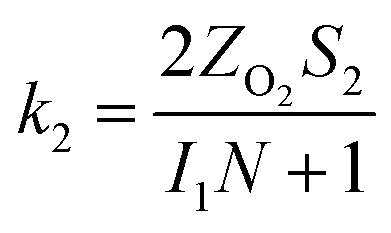
8
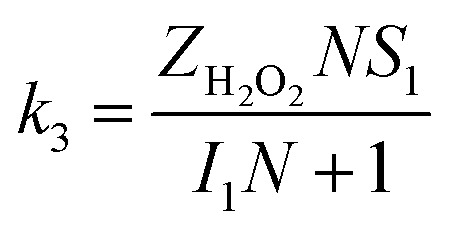
9
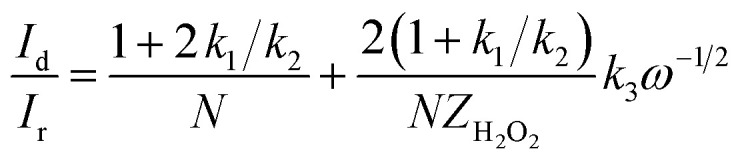
10
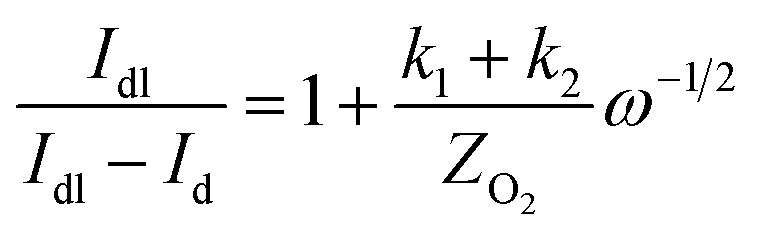
*Z*_H_2_O_2__ = 0.62*D*_H_2_O_2__^2/3^*ν*^−1/6^ and *Z*_O_2__ = 0.62*D*_O_2__^2/3^*ν*^1/6^, where *D*_H_2_O_2__ and *D*_O_2__ are diffusion coefficients of H_2_O_2_ and O_2_, respectively. *I*_1_ and *S*_1_ are the intercept and slope from the plot of *I*_d_/*I*_r_*vs. ω*^−1/2^ shown in eqn (9), and *S*_2_ is the slope from the plot of *I*_dl_/(*I*_dl_ − *I*_d_) *vs. ω*^−1/2^ shown in eqn (10). *I*_dl_ is the disk-limiting current.^41^

**Fig. 8:**
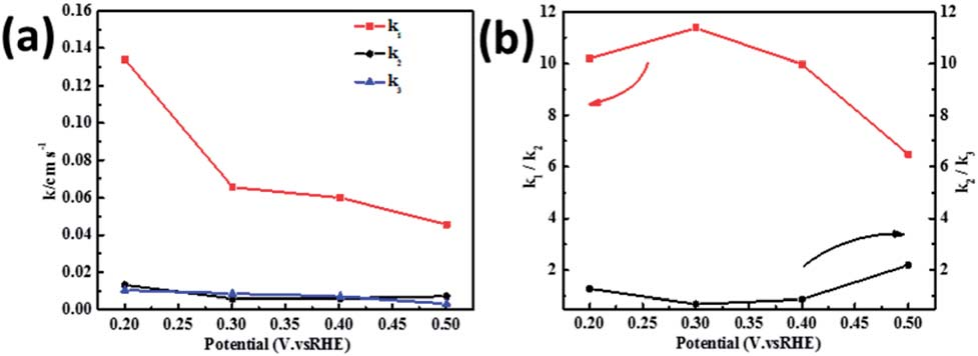
(a) *k*_1_, *k*_2_, and *k*_3_ values of PPYTZ–Fe/C and (b) values of *k*_1_/*k*_2_ and *k*_2_/*k*_3_.

Fig. 8 shows the values of *k*_1_, *k*_2_, and *k*_3_ and the ratio of *k*_1_/*k*_2_, *k*_2_/*k*_3_ for the PPYTZ–Fe/C catalysts, respectively. The data shows that the reaction rate of the PPYTZ–Fe/C catalyst is *k*_1_ ≫ *k*_2_, *k*_3_ = 0, indicating that the oxygen reaching the electrode is reduced to H_2_O directly. The high value of *k*_1_/*k*_2_ is almost 10, leading to higher electron transfer numbers and lower H_2_O_2_ values. These results indicate that the ORR process of PPYTZ–Fe/C catalyst is mainly *via* four-electron transfer routes consisting of a direct four-electron route.

## Conclusions

In summary, we prepared two catalysts, namely PPYTZ/C and PPYTZ–Fe/C. PPYTZ–Fe/C is a new type of Fe–N–C material, which is chemically polymerized by 2,6-pyridinedicarboxylic acid chloride and 3,5-diamino-1,2,4-triazole as a precursor, and iron ions were introduced and loaded onto the carbon powder. Compared with the PPYTZ/C catalyst, PPYTZ–Fe/C processed higher oxygen reduction performance and faster catalytic kinetics. The ORR process intended to follow a four-electron pathway compared to the two-electron pathway of PPYTZ/C. The limit current and stability was greatly improved, and the hydrogen peroxide yield was decreased on PPYTZ–Fe/C. PPYTZ–Fe/C was rich in Fe–N coordination sites and exhibited good ORR properties in alkaline media. Thus, it could be used as a potential fuel cell cathode catalyst in future.

## Conflicts of interest

There are no conflicts to declare.

## Supplementary Material

RA-010-C9RA09301J-s001
