# Supplementary material for: Insights into the role of an Fe–N active site in the oxygen reduction reaction on carbon-supported supramolecular catalysts
Source: RSC Adv. 2020 Feb 28;10(15):8709–16. doi: 10.1039/c9ra09301j (PMC9050072; doi:10.1039/c9ra09301j)
Supplement: RA-010-C9RA09301J-s001 [file RA-010-C9RA09301J-s001.pdf]

Electronic Supplementary Material (ESI) for RSC Advances.
This journal is © The Royal Society of Chemistry 2020

Insights into the role of Fe-N active site in the oxygen reduction reaction on carbon-supported supramolecule catalysts

Lin Gu[a], Yunyun Dong[b, *], Yan Zhang[a], Bo Wang[b], Qing Yuan[b], Hongmei Du[a], Jinsheng Zhao[a, *]

[a]Shandong Key Laboratory of Chemical Energy Storage and Novel Cell Technology, Liaocheng University, Liaocheng, 252059, P.R China.

[b]College of Chemistry and Chemical Engineering, Liaocheng University, Liaocheng, 252059, P.R. China.

The characterizaton of PPYTZ polymer

The ^{1}H NMR (δ ppm) and IR spectroscopy of PPYTZ polymer are shown in Fig. 1S. Wherein, the detailed analysis of the ^{1}H NMR (Fig. 1Sa) is as follows δ: ^{1}H NMR(400 MHz, $(CD_3)_2SO$, δ ppm) δ=13.71(s, 7H), 12.77(s, 10H), 11.61(s, 11H), 8.42(t, 12H), 8.32(m, 23H), 5.81(s, 6H), 3.06(q, 1H), 1.34(s, 1H), 1.22(d, 3H), 1.16(t, 1H). In addition, Fig. 1Sb shows the data of the IR : the peak at 1692 cm^{-1} is due to the CO-NH stretching vibration [1], the bands at 1538 cm^{-1} and 1457 cm^{-1} are attributed to stretching vibration of pyridine ring (C=C and C=N) [2], the peak at 1296 cm^{-1} is due to N-N stretching vibration of triazole ring, the bands in the region 907-644 cm^{-1} are assigned to C-H in-of-plane bending vibration of aromatic ring [3]. Therefore, he above data analysis shows that the PPYTZ polymer has been successfully prepared

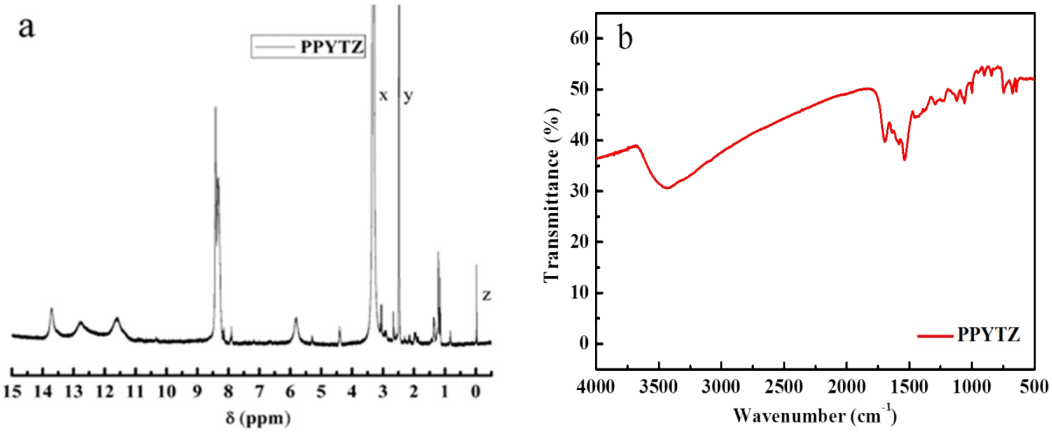

Fig. 1S ^{1}H NMR (a) and IR (b) of PPYTZ polymer

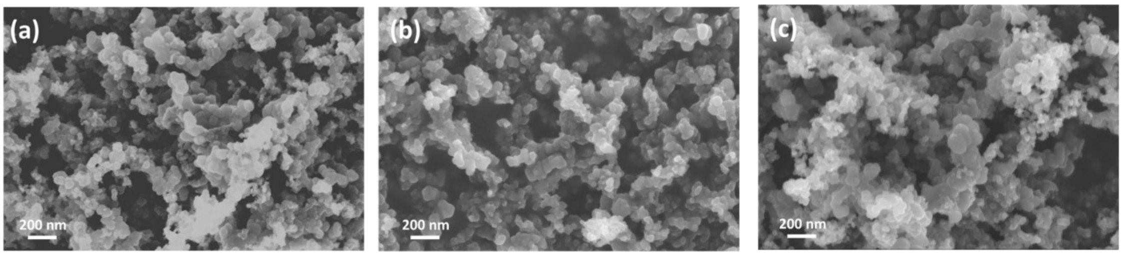

Fig. 2S SEM images of (a) Vulcan XC-72, (b) PPYTZ/C and (c) PPYTZ-Fe/C.

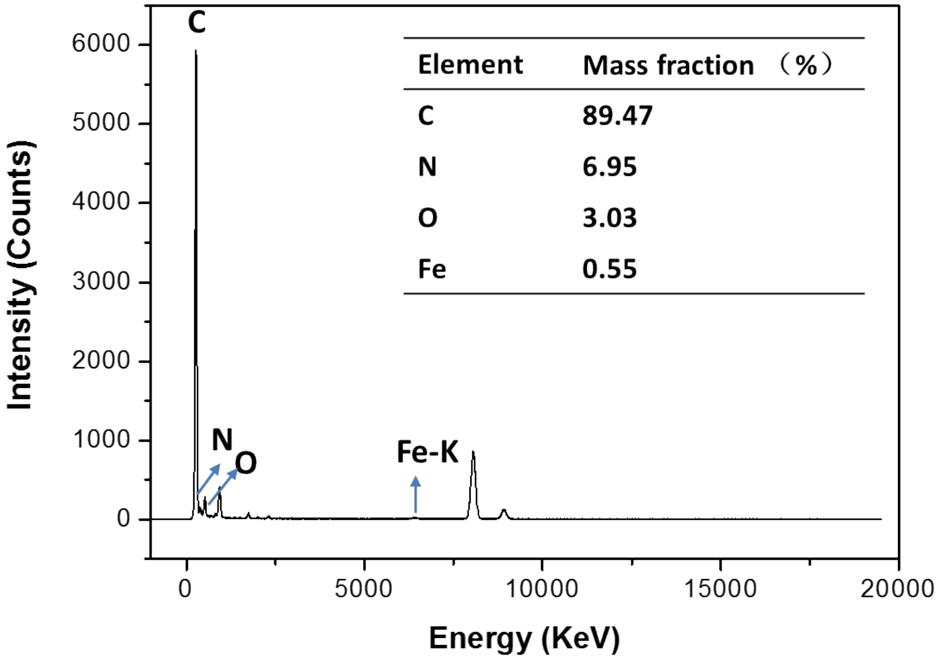

Fig. 3S TEM-EDS image of PPYTZ-Fe/C.

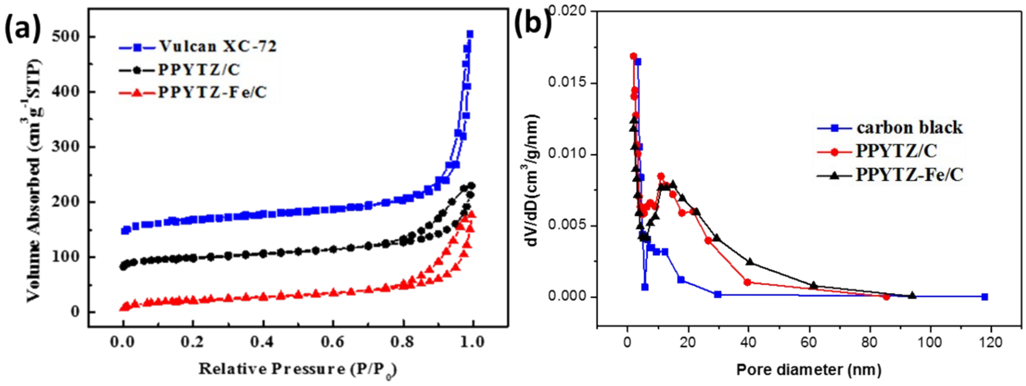

Fig. 4S N_2 adsorption/desorption isotherms and BJH pore size distribution data of Vulcan XC-72, PPYTZ/C and PPYTZ-Fe/C composite.

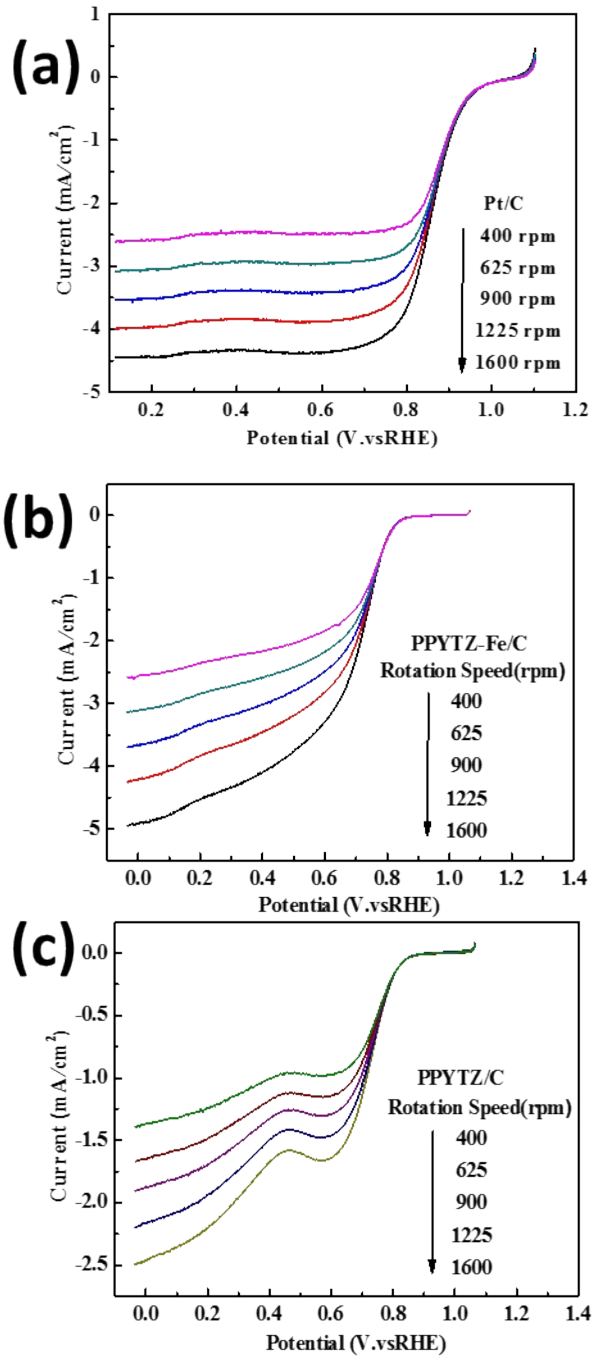

Fig. 5S (a) Pt/C, (b) PPYTZ/C and (c) PPYTZ-Fe/C polarization curves at different speeds in 0.1M KOH . Scan rate: 10 mV $\mathbf{s^{-1}}$**.**

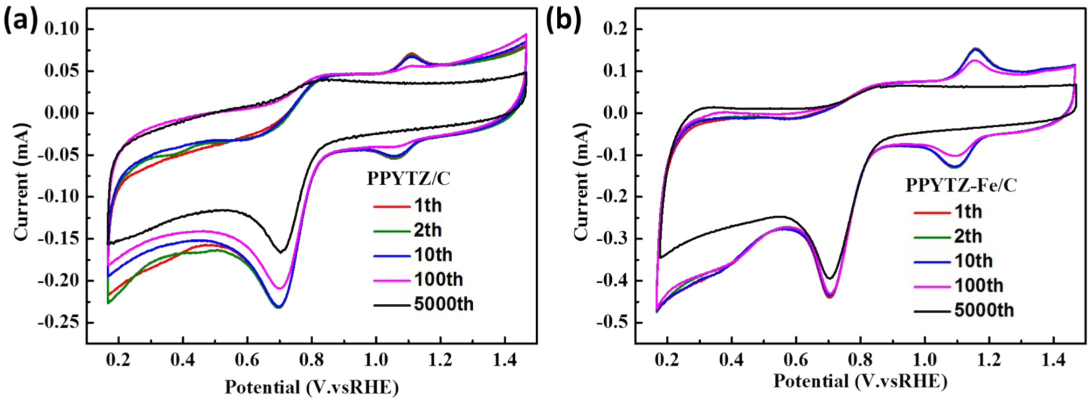

Fig. 6S 5000th CV curves of (a) PPYTZ/C and (b) PPYTZ-Fe/C in O_2 saturated KOH (0.1M) solution.
